# Acute Communication Between Microglia and Nonparenchymal Immune Cells in the Anti-Aβ Antibody-Injected Cortex

**DOI:** 10.1523/JNEUROSCI.1456-24.2024

**Published:** 2024-12-31

**Authors:** Kate E. Foley, Erica M. Weekman, Katelynn E. Krick, Sherika N. Johnson, Tiffany L. Sudduth, Donna M. Wilcock

**Affiliations:** ^1^Stark Neurosciences Research Institute, Indiana University School of Medicine, Indianapolis, Indiana 46202; ^2^Neurology, School of Medicine, Indiana University School of Medicine, Indianapolis, Indiana 46202; ^3^Anatomy, Cell Biology, and Physiology, Indiana University School of Medicine, Indianapolis, Indiana 46202; ^4^Sanders-Brown Center on Aging, Department of Physiology, University of Kentucky, Lexington, Kentucky 40536

**Keywords:** Alzheimers disease, amyloid, antiamyloid antibody, inflammation, microglia, single-cell sequencing

## Abstract

Anti-Aβ immunotherapy use to treat Alzheimer's disease is on the rise. While anti-Aβ antibodies provide hope in targeting Aβ plaques in the brain, there still remains a lack of understanding regarding the cellular responses to these antibodies in the brain. In this study, we sought to identify the acute effects of anti-Aβ antibodies on immune responses. To determine cellular changes due to anti-Aβ antibody exposure, we intracranially injected 14 mo APP male and female mice with anti-Aβ IgG1 (6E10) or control IgG1 into the cortex. After 24 h or 3 d, we harvested the cortex and performed a glial cell-enriched preparation for single-cell sequencing. Cell types, proportions, and cell-to-cell signaling were evaluated between the two injection conditions and two acute timepoints. We identified 23 unique cell clusters including microglia, astrocytes, endothelial cells, neurons, oligos/OPCs, immune cells, and unknown. The anti-Aβ antibody-injected cortices revealed more ligand–receptor (L–R) communications between cell types, as well as stronger communications at only 24 h. At 3 d, while there were more L–R communications for the anti-Aβ antibody condition, the strength of these connections was stronger in the control IgG condition. We also found evidence of an initial and strong communication emphasis in microglia-to-nonparenchymal immune cells at 24 h, specifically in the TGFβ signaling pathway. We identify several pathways that are specific to anti-Aβ antibody exposure at acute timepoints. These data lay the groundwork for understanding the brain's unique response to anti-Aβ antibodies.

## Significance Statement

Anti-Aβ monoclonal antibody therapy is the first disease-modifying therapy to be traditionally approved by the FDA. The target population is relatively small due to the incidence of adverse vascular reactions in a significant proportion of individuals. While these adverse reactions are prevalent, our basic understanding of the cellular responses to these antibodies limits our ability to further refine this therapeutic approach to increase safety and accessibility. These studies are the first, but critical, step in understanding the brain's response to anti-Aβ antibodies.

## Introduction

Alzheimer's disease (AD) is a leading cause of dementia affecting approximately 10.8% of Americans over the age of 65 and 6.7 million Americans total (Alzheimer's Association, 2023). There are significant efforts to develop disease-modifying therapies to mitigate AD pathology and subsequently slow or prevent cognitive decline. Anti-Aβ immunotherapies, such as aducanumab, lecanemab, and donanemab, lower parenchymal amyloid burden in clinical trials. Lecanemab, an anti-Aβ targeting monoclonal antibody, received traditional FDA approval after showing clinical trial data that revealed a significant slowing of cognitive decline by approximately 30% compared with the placebo treatment (Budd Haeberlein et al., 2022; Van Dyck et al., 2022; Sims et al., 2023). More recently, donanemab, an antibody targeting pyroglutamate-modified Aβ found in plaques and cerebral amyloid angiopathy (CAA), also received traditional FDA approval with a similar slowing of cognitive decline. Consistent findings of cerebrovascular adverse events, deemed amyloid-related imaging abnormalities (ARIA), continue to plague these therapeutic approaches which restrict who should and should not receive the drugs and also complicate the clinical workflows of screening and administration. Now in clinical use in AD patients across the United States and other countries (Release FN, 2023a,b; Wu et al., 2023), there still remains a lack of clear understanding of the molecular mechanisms underlying both the plaque clearance and ARIA (Pfeifer et al., 2002; Sperling et al., 2011; Salloway et al., 2022).

Decades of research have led to our current understanding of the clear influence that the native immune system has in the brain, primarily comprised of microglia, altering Aβ plaque clearance, especially anti-Aβ antibody-mediated Aβ plaque clearance (Spangenberg et al., 2019; Kiani Shabestari et al., 2022; De Schepper et al., 2023; Cadiz et al., 2024). Originally considered immune privileged and incapable of the immune responses seen in the periphery, we now understand that the resident microglia have the capacity to respond to stimuli in complex and diverse ways. With the advent of single-cell sequencing technologies, we are able to identify various microglia cell states and transitions, loosely indicating the priorities of the cell. Combined with our enhanced understanding of the nonparenchymal immune cells can play in AD and their role in the regulation of Aβ conformations and levels, microglia are hypothesized to be critical mediators of both plaque clearance and cerebrovascular dysfunction due to anti-Aβ antibody therapy (Rogers, 2023; Taylor et al., 2023).

In the current study, we proposed that the anti-Aβ antibody would influence the immune cell states of microglia and nonparenchymal immune cells immediately upon exposure to anti-Aβ antibodies. Studies from 20 years ago showed that intracranial anti-Aβ antibody injections elicit significant diffuse plaque clearance in the cortex and hippocampus at 24 h and significant compact plaque clearance after 3 d when compared with the contralateral IgG antibody-injected side (Wilcock et al., 2003). Furthermore, microglial immunoreactivity only occurred after 3 d, despite the early diffuse plaque clearance, suggesting a possible microglia-independent clearance mechanism (Wilcock et al., 2003). We sought to leverage new technologies and our updated understanding of neuroinflammation to evaluate how microglia and other immune cells are responding. We performed single-cell RNAseq to differentiate specific effects that the anti-Aβ antibody has on transcriptional profiles of microglia and immune cells over these same time periods, 24 h and 3 d. We found that microglial and other immune cell crosstalk is influenced by an acute anti-Aβ antibody exposure in the brain, indicating anti-Aβ antibody-specific cell-to-cell communication occurring in key inflammation pathways.

## Materials and Methods

### Mouse husbandry

Male and female APP mice (Tg2576, APPSwe) were maintained at the University of Kentucky (UKY) following Institutional Animal Care and Use Committee protocols. All mice were fed ad libitum and kept on a 12 h light/dark cycle. Mice were aged 14 months and underwent intracranial injection surgery.

### Intracranial injection surgery and euthanasia

All mice were randomized and assigned to mouse monoclonal (1) anti-Aβ IgG1κ (6E10; BioLegend 803001) targeting residues 1–16 of the Aβ peptide or (2) control IgG_1_κ isotype control (R&D Systems; MAB002) injection condition and harvest timepoint (24 h or 3 d post intracranial injection). Randomization was balanced for sex effects; however, this study was not powered to test for the effect of sex. Intracranial injections utilized a Stoelting stereotaxic system (51733D), and injections were placed bilaterally in the cortex following previously published protocols (Weekman et al., 2014). After induction of anesthesia, mice were transferred to the stereotaxic frame, 2 µl of 1 mg/ml of 6E10 or IgG1 was injected over a 4 min period, and the incision was stapled until harvest. Beuthanasia-D was administered at a lethal dose, and each mouse underwent gravity perfusion with 20 ml of normal saline.

### Single-cell suspension preparation

Mice were randomly selected but balanced for sex (M vs F), injection condition (anti-Aβ vs control IgG), and timepoint (24 h vs 3 d) for single-cell sequencing. Each of the four groups (Control_24 h, Control_3 d, anti-Aβ_24 h, and anti-Aβ_3 d) contained three mice, each with two males and one female except for the Control_24 h condition which had two females and one male. After gravity perfusion, brains were carefully removed, bisected on ice, and the cortex removed from the left half and prepared for single-cell suspension protocols following the Miltenyi Adult Brain Dissociation Kit (ABDK) protocol and previous publications with minor modifications (Early et al., 2020). Once removed, cortical tissue was minced and exposed to the ABDK enzymatic mixes prior to octodissociation. Substantial effort was taken to minimize time spent at room temperature, with approximately 15 min or fewer from the start of mouse perfusion to the start of the Miltenyi octodissociation (37C_ABDK_01 program) protocols. After octodissociation, all samples were strictly maintained on ice or centrifuged at 4°C, to maintain RNA integrity. Tissue suspension was collected, and the C-tube was washed with 5 ml cold DPBS to ensure majority of tissue transfer. Suspension was strained through 70 µm strainers, washed in 5 ml cold DPBS, and centrifuged (10 min, 300 × *g*, accel = 9, decel = 5). Supernatant was discarded, pellet was resuspended in 3.1 ml cold DPBS and mixed, and then 900 µl Miltenyi debris removal solution was added. An additional 4 ml cold DPBS was overlayed carefully, and suspension was centrifuged again (3,000 × *g*, 10 min, accel = 9, decel = 0). Clear top supernatant and debris layer was removed and discarded and cold DPBS until 15 ml was added, inverted multiple times for mixing, and strained through a 30 µm strainer. Suspension was centrifuged again (1,000 × *g*, 10 min, accel = 9, decel = 9), and supernatant was removed. Pellet was resuspended in 250–300 µl DPBS plus BSA and transported quickly on ice for cell viability evaluation and partitioning.

### Cell partitioning, sequencing, and sequence processing

Cell viability for the 12 samples was over 93% except for two samples where cell viability was 70 and 86%. The Chromium platform at the University of Kentucky Arts & Sciences Imaging Center was used for partitioning and sequencing preparation according to the manufacturer's protocols (10x Genomics 3′ Gene Expression Dual Index Kit; index type, i7 and i5; index length, 10 nt; insert size, 350 bp). Each partitioned sample captured 10,000 cells for sequencing. RNA sequencing was performed by Novogene using all 12 individual samples and two NovaSeq lanes (paired end 150 × 2). Raw fastq sequencing data for each sample were processed using CellRanger and mouse reference genome GRCm38 (mm10).

### Single-cell sequencing analysis

**Figure 1.**
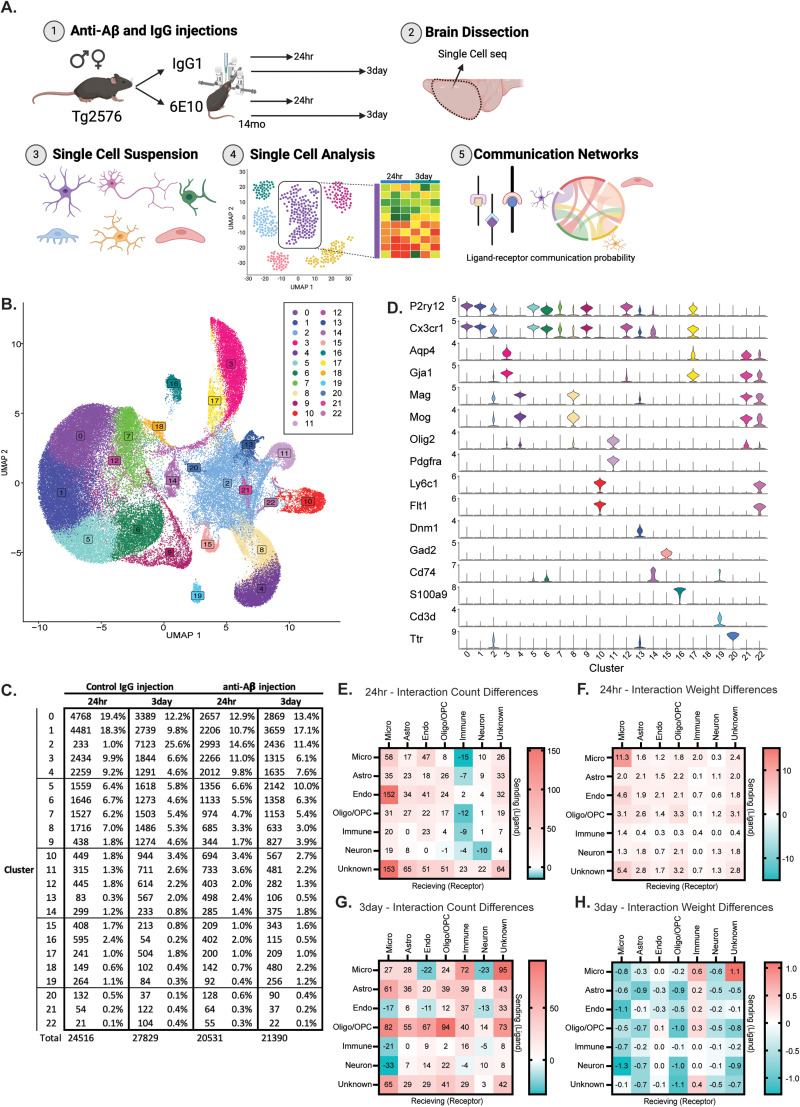
Anti-Aβ antibody alters cell–cell communication networks. ***A***, A schematic of study design and analysis. ***B***, The UMAP depicting 23 unique clusters found from glial-enriched single-cell sequencing prep and analysis. UMAPs for sample, condition, sex, and timepoint can be found in Extended Data Figure 1-1. There were no preparation batch or sex-specific differences noted (Extended Data Table 1-1). ***C***, The number of cells and percentage of total cell population for each cluster. ***D***, Marker genes of various cell types to aid in identification of cluster cell type identity (Exteded Data Table 1-2). ***E***, The difference in the number of significant ligand–receptor pairing counts (Aβ-IgG) at 24 h postinjection. Sending (ligand) cell types are along the *y*-axis/left, while receiving (receptor) cell types are along the *x*-axis/top. Red indicates more interactions in the Aβ injection condition, and blue indicates more interactions in the IgG injection condition. ***F***, The difference in weight/strength of the significant ligand–receptor interactions (Aβ-IgG) at 24 h postinjection. ***G***, The difference in the number of significant ligand–receptor communication counts (Aβ-IgG) at 3 d postinjection. ***H***, The difference in weight/strength of the significant ligand–receptor interactions (Aβ-IgG) at 3 d postinjection.

R (v4.3.2) was used to run Seurat (v5.0.1), and we performed the Seurat package recommended quality control with minor adjustments (Hao et al., 2023). Filtered CellRanger outputs for each sample (barcodes.tsv.gz, features.tsv.gz, and matrix.mtx.gz) were localized into 12 individual folders named per sample name and uploaded into R using the “Read10×” command. To aggregate all samples into one object, we used Seurat's “CreateSeuratObject.” Cells were filtered out if they had (1) under 200 or over 5,000 unique features and if they (2) had over 5% mitochondrial counts. Counts were normalized using the “LogNormalize” method and a default scale factor of 10,000. Then, the “FindVariableFeatures” command was run using variance stabilized transformation (vst) and a default of 2,000 features, and then the data were scaled using the “ScaleData” function on all genes. Principle component analysis (PCA) was next used to visualize the dimensionality of the data, and the first 11 principal components (interpreted by “elbowplot” drop-off as recommended) were inputted into the “FindNeighbors” function. Next “FindClusters” was run using a 0.5 resolution, and 23 unique clusters were identified. A UMAP graph was created for visualization and parsing out cell clusters (Fig. 1*B*). Total cells analyzed for each condition were compared and were similar between injections and timepoints. From this, the percentage of cells per cluster was calculated based on all cells per injection and timepoint (Fig. 1*C*).

10.1523/JNEUROSCI.1456-24.2024.t1-1 Table 1-1 Download Table 1-1, XLSX file.

10.1523/JNEUROSCI.1456-24.2024.t1-2 Table 1-2 Download Table 1-2, XLS file.

10.1523/JNEUROSCI.1456-24.2024.f1-1 Figure 1-1 UMAP graphs colored for injection type, timepoint, sex, and sample ID. Download Multimedia/Extended Data, TIF file.

We next evaluated each cluster for key marker genes from multiple literature searches and previously established single-cell markers (Fig. 1*D*; Keren-Shaul et al., 2017; Yang et al., 2021; Marsh et al., 2022). For this paper, violin plots of gene expression per cluster were created using the “Stacked_VlnPlot” from the scCustomize package (v2.0.1). Cluster identities were further confirmed by the PanglaoDB_Augmented_2021 database through enrichR (v3.2; data not shown, Fig. 3*B*). Cluster percentage charts were created outside of R using Prism (v10.0.3).

CellChat (v1.6.1) was utilized to identify outgoing and incoming communications between clusters and cell types (Jin et al., 2021a). Briefly, Seurat data were split by timepoint (24 h_antiAB vs 24 h_IgG, and 3 _antiAB vs 3 d_IgG) for more in-depth analysis between injection conditions. “netAnalysis_computeCentrality” was run on each set individually before merging into one CellChat object per timepoint. While multiple cell type subtypes were found (i.e., microglia clusters C0, C1, C5, C6, C7, C9, and C12), we simplified the cell-type categories into their more major cell type name (i.e., “Micro”) for further downstream analysis. Interaction count and weight differences (Fig. 1*E–H*) were derived from “netVisual_diffInteraction” by instead pulling data from the saved source using object.list[[1]]@net$count.merged, after which subtractions were calculated and visualization into a heatmap graphic was made for ease of viewing using Prism. Other data visualization was created by changing arguments in “rankNet,” “netVisual_aggregate” (using the “chord” layout), and the “netAnalysis_signalingRole_scatter” functions within the CellChat package.

### Data and code availability

Raw data will be deposited onto Synapse Platform (Sage Bionetworks), as well as on NCBI GEO upon acceptance of this manuscript. Code utilized in R is uploaded to GitHub including modified Seurat code with visualization, CellChat code for 24 h comparisons, and CellChat code for 3 d comparisons (https://github.com/kefoley06/Acute_responses_to_anti-AB_antibody).

## Results

### Determining cluster identity and cell proportion differences between injection and timeframe

To identify transcriptional changes in various cell types due to anti-Aβ antibody, we injected commercial 6E10 anti-Aβ IgG1 antibody or a control IgG1 antibody into the anterior region of the cortex of 14-month-old male and female APP mice (Fig. 1*A*, see Materials and Methods). The anterior half of the cerebral cortex was harvested 24 h (24 h) or 3 d (3 d) after intracranial injection and prepared using a glial-enriched isolation protocol for subsequent single-cell sequencing. Twenty-three unique clusters were identified, and the number of cells per cluster for each injection and timepoint was identified (Fig. 1*B*). We also calculated the proportion of cells per cluster (Fig. 1*B,C*). Key cell marker genes aided in identifying cell types identifying microglia (0, 1, 5, 6, 7, 9, and 12), astrocytes (3, 17), endothelial cells (10, 22), neurons (13, 15), oligodendrocytes and precursor cells (OPCs; 4, 8, 11), nonparenchymal immune cells (immune; 14, 16, 19), and unknown (2, 18, 20, 21; Fig. 1*D*). Cell-to-cell communication was evaluated using CellChatDB, revealing an overall increase in number of ligand–receptor (L–R) counts at 24 h and 3 d for the anti-Aβ antibody condition (Fig. 1*E,G*), with increased strength for anti-Aβ antibody injected communications at 24 h but increased strength for IgG antibody at 3 d (Fig. 1*F,H*). Interestingly, the greatest communication strength was among the microglia, with microglia sending and receiving communications 24 h after an anti-Aβ antibody injection (Fig. 1*F*). While there were modestly more interactions between microglia at the 24 h timepoint in the anti-Aβ antibody injected condition, these communications were significantly stronger. These data suggest that after only 24 h of antibody presence in the brain there is an acute communication shift between multiple cell types in response to anti-Aβ IgG1 antibody compared with a nonspecific IgG1 antibody.

### Anti-Aβ antibody triggers strong inflammatory signaling after 24 h

**Figure 2.**
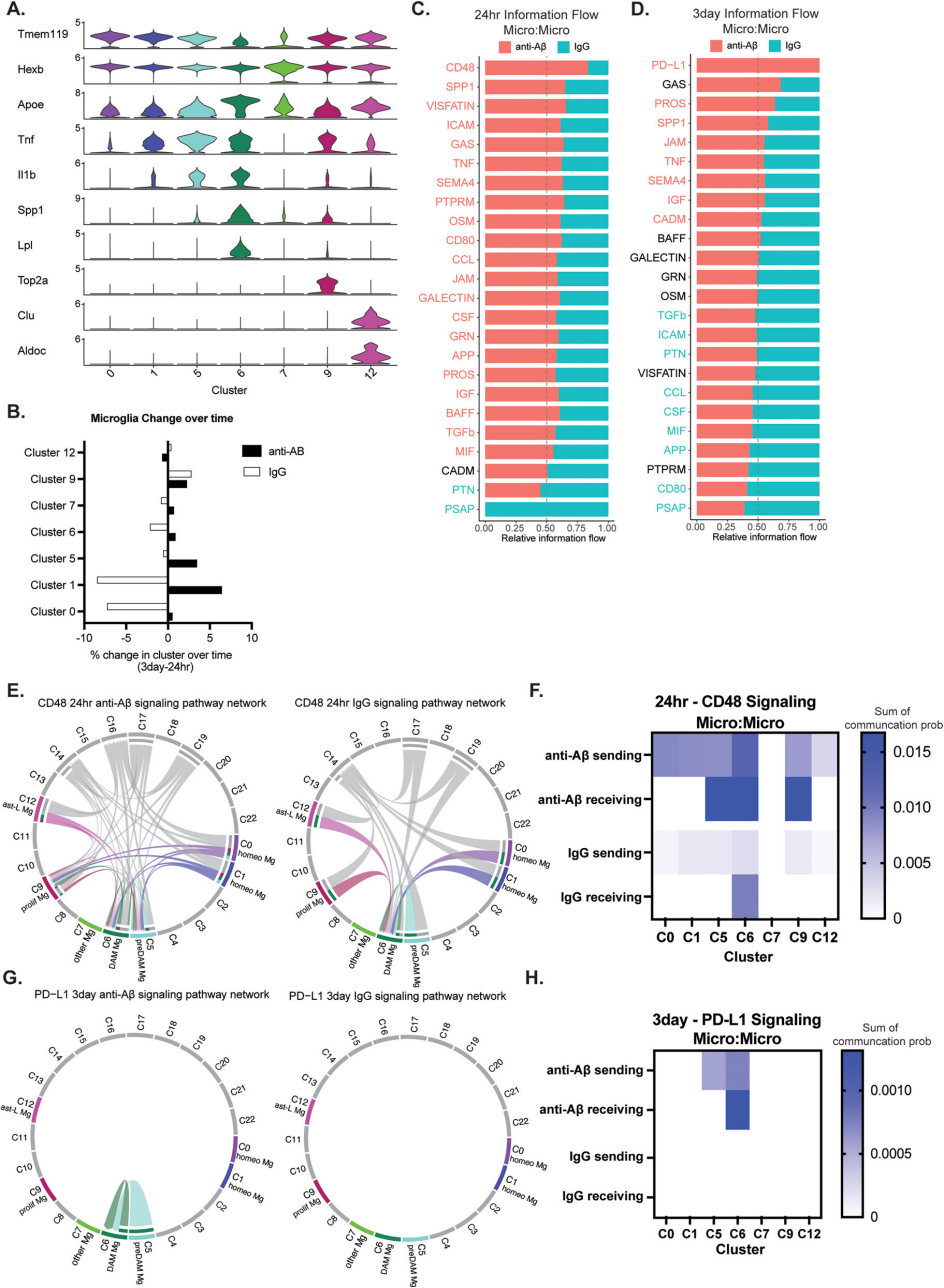
Microglia show a temporal change in communication due to anti-Aβ antibody exposure. ***A***, Marker genes to identify subtypes of various microglia states. ***B***, Percent change in microglia cells per cluster from 24 h to 3 d (Extended Data Fig. 2-1). ***C***, Signaling pathways enriched in micro (sending):micro (receiving) communication at 24 h in anti-Aβ (pink) and IgG (teal). ***D***, Signaling pathways enriched in micro (sending):micro (receiving) communication at 3 d in anti-Aβ (pink) and IgG (teal). ***E***, A chord diagram of significant CD48 signaling at 24 h in Aβ injected (left) and IgG injected (right). ***F***, The sum of sending and receiving communication probability per cluster for CD48 signaling at 24 h. ***G***, A chord diagram of significant PD-L1 signaling at 3 d in Aβ injected (left) and IgG injected (right). ***H***, The sum of sending and receiving communication probability per cluster for PD-L1 signaling at 3 d. Extended Data Figure 2-2 shows CCL signaling and SPP1 signaling results. Microglia communications are colored according to cluster sending (ligand), and nonmicroglia communications are in gray. Statistically significant communication terms are noted with their respective colors (Wilcoxon test, *p* < 0.05). Mg, microglia; homeo, homeostatic; pre-DAM, pre-disease-associated microglia; DAM,¸disease-associated microglia; prolif, proliferative; ast-L, astrocyte-like.

Due to the number and strength of the microglial immune response, we next queried which microglia communications were prioritized after anti-Aβ antibody injection at 24 h and 3 d. First, we established which microglia subtypes were present to more discretely determine communication differences at different cell states. Examination of microglial genes revealed various microglia states that have previously been reported on in the literature but not how they change with anti-Aβ antibody: homeostatic (0, 1), pre-disease-associated microglia (DAM; 5), DAM (6), proliferative (9), astrocyte-like (12), and other (7; Fig. 2*A*). General shifts in the microglial cluster populations were observed. Microglial cluster proportions increased between 24 h and 3 d in the anti-Aβ antibody-injected group, and there were fewer microglia per cluster between 24 h and 3 d in the IgG1 antibody condition (Fig. 2*B*). In the anti-Aβ antibody injected condition, there was a general increase in microglia percentage from 24 h to 3 d. In the IgG antibody injected condition, there was a general decrease in microglia percentages between 24 h and 3 d, suggesting a differential shift of the number of cells in these microglia clusters in response to anti-Aβ antibody and IgG antibody. In contrast, we found an increase in cells captured in clusters 0 (homeostatic), 1 (homeostatic), 5 (pre-DAM), 6 (DAM), 7 (other), and 9 (proliferative) after anti-Aβ antibody injection at 3 d. Importantly, cluster size is controlled for in CellChat’s communication algorithm, preventing the total population from weighing into the ligand–receptor analysis and total information flow (Jin et al., 2021).

10.1523/JNEUROSCI.1456-24.2024.f2-1 Figure 2-1 Percentage of cells in microglia clusters at 24hrs and 3days. Download Multimedia/Extended Data, TIF file.

10.1523/JNEUROSCI.1456-24.2024.f2-2 Figure 2-2 A. Chord plot of CCL signaling at 24hr in the anti-Aβ antibody and IgG antibody conditions.B. Sum of sending and receiving communication probability per cluster for CCL signaling at 24hr between microglia.C. Chord plot of CCL signaling at 3day in the anti-Aβ antibody and IgG antibody conditions.D. Sum of sending and receiving communication probability per cluster for CCL signaling at 3day between microglia.E. Chord plot of SPP1 signaling at 24hr in the anti-Aβ antibody and IgG antibody conditions.F. Sum of sending and receiving communication probability per cluster for SPP1 signaling at 24hr between microglia.G. Chord plot of SPP1 signaling at 3day in the anti-Aβ antibody and IgG antibody conditions.H. Sum of sending and receiving communication probability per cluster for SPP1 signaling at 3day between microglia.
Microglia communications are colored according to cluster sending (ligand), and non-microglia communications are in grey. Download Multimedia/Extended Data, TIF file.

We next examined the information flow among microglia (total weights in the signaling pathway network) between the anti-Aβ antibody and IgG antibody injected conditions at 24 h (Fig. 2*C*), and 3 d (Fig. 2*D*). These plots show the total communication probability (weights) of many signaling pathways between the designated cell populations (here, microglia:microglia) and whether they are significantly enriched in either the anti-Aβ antibody (pink) or IgG antibody control condition (teal; Jin et al., 2021). There was a substantial increase in the number of enriched signaling pathways for the anti-Aβ antibody condition at 24 h compared with the IgG1 antibody condition including the “CD48,” “SPP1,” “TNF,” “APP,” and “TGFβ” pathways. Interestingly, the 24 h specific CD48 signaling showed ligand (sending) signaling across microglial clusters 0, 1, 5, 6, 9, and 12, in both injection conditions; however, the strength of these sending signals was much stronger in the anti-Aβ antibody injected condition (Fig. 2*E,F*). Similarly, the CD48 receptor (receiving, Cd244a) was found to be probable across microglia clusters 5, 6, and 9 in the anti-Aβ antibody condition, while only in the DAM cluster 6 in the IgG antibody condition. Several pathways were enriched at both 24 h and 3 d after anti-Aβ antibody injections (e.g., SPP1, SEMA4, IGF, and JAM signaling). While communications revealed increased micro-to-micro signaling at 24 h, 3 d postinjection revealed both anti-Aβ and IgG1 antibodies contained multiple significantly enriched signaling pathways, suggesting that the microglial cellular communication response to IgG1 antibody backbone may take longer to initiate. ICAM, CD80, CCL, CSF, APP, TGFβ, and MIF signaling showed a temporal switch in significant enrichment conditions. “CCL” signaling included many chemokines and complement receptors (CCRs) which were shown to be both sending and receiving signals in almost all microglia subtypes (except cluster 7). The strength of these connections, however, shifted over the course of 24 h and 3 d, where the connections were stronger in the anti-Aβ antibody group at 24 h and stronger in the IgG1 antibody injection group at 3 d. The newly enriched PD-L1 signaling was seen only at 3 d and was only enriched in the anti-Aβ antibody condition. This could suggest a possible immune exhaustive phenotype for the DAM cluster 6 cells (Fig. 2*G,H*).

### Examination of perivascular macrophages and nonparenchymal immune cells response to anti-Aβ injection

**Figure 3.**
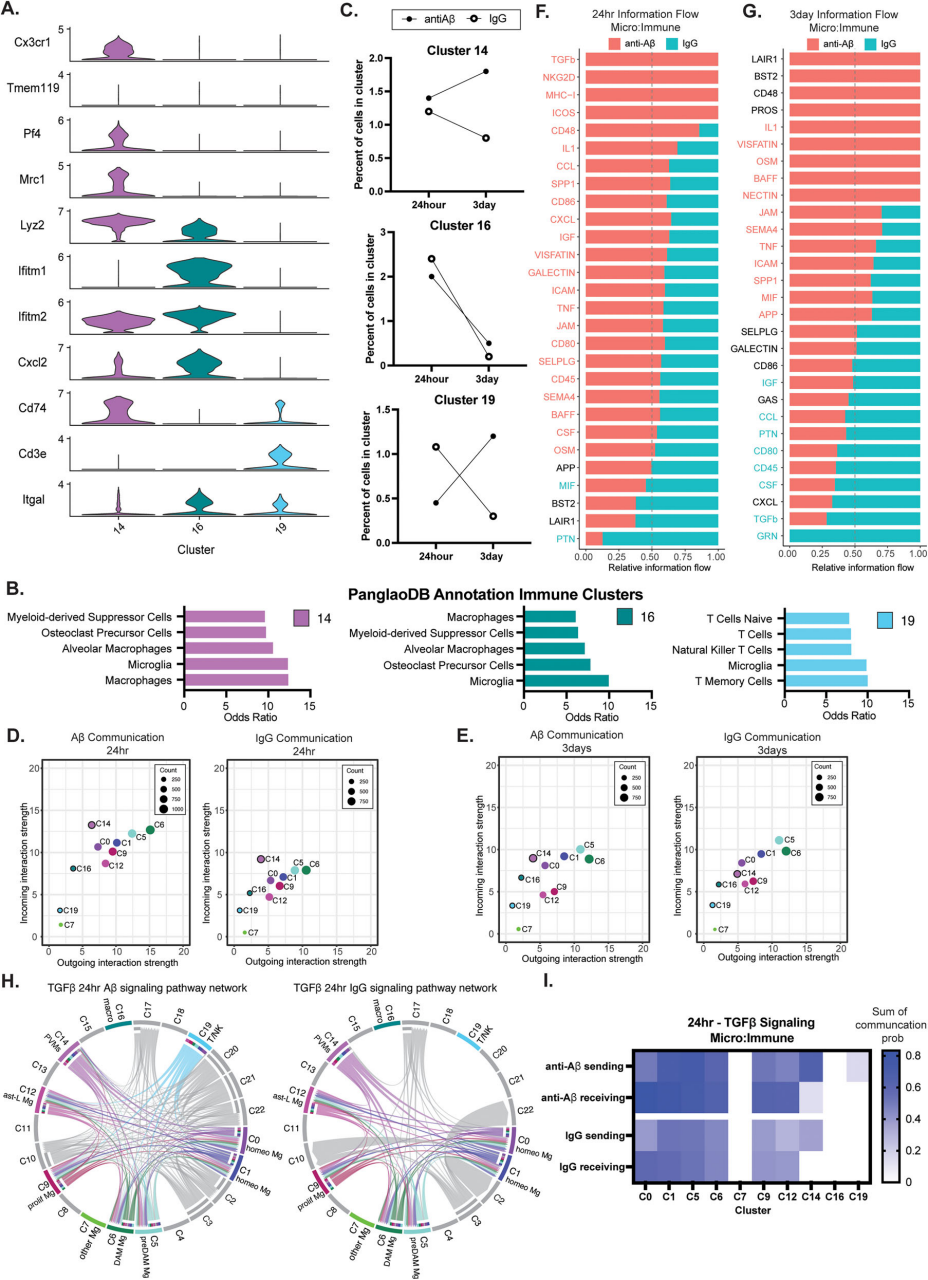
Microglia signal nonmicroglial immune cells through TGFβ. ***A***, Immune cell cluster identification through multiple marker genes. Cluster 14 expresses multiple perivascular macrophage genes, cluster 16 expresses some PVM and high response to interferon response genes suggesting a macrophage cell type, and cluster 19 expresses markers for T-cells and NK cells. ***B***, The PanglaoDB annotation of immune cell clusters graphed by odds ratio for the top five likely cell types (Extended Data Fig. 3-1). ***C***, Percent changes in the number of immune cells in anti-Aβ and IgG injected conditions between 24 h and 3 d. ***D***, The overall outgoing and incoming communication interaction strength for microglia and nonparenchymal cell clusters in anti-Aβ-injected and IgG-injected mice at 24 h (3 d; Extended Data Fig. 3-1). The black outline differentiates nonmicroglia immune cell clusters, and no outline indicates a microglia cluster. ***E***, The overall outgoing and incoming communication interaction strength for microglia and nonparenchymal cell clusters in anti-Aβ- and IgG-injected mice at 3 d. The black outline differentiates nonmicroglia immune cell clusters, and no outline indicates a microglia cluster. ***F***, The signaling pathways enriched in micro (sending):immune (receiving) communication at 24 h in anti-Aβ (pink) and IgG (teal). ***G***, The signaling pathways enriched in micro (sending):immune (receiving) communication at 3 d in anti-Aβ (pink) and IgG (teal). ***H***, A chord diagram of significant TGFβ signaling at 24 h in Aβ-injected (left) and IgG-injected (right). ***I***, The sum of sending and receiving communication probability per immune cluster for TGFβ signaling at 24 .

Though micro-to-micro communication was highlighted, our results also showed communication between microglia and the nonparenchymal immune cell clusters. There is a suggestion in the literature that nonparenchymal immune cells play important roles in anti-Aβ immunotherapy responses; therefore, we next evaluated the signaling pathways between microglia and immune cells (Taylor et al., 2023). Key marker genes helped identify cluster 14 as perivascular macrophages and cluster 19 as T-cells/natural killer (NK) cells, while cluster 16 contained no markers of residential microglia and some expression of perivascular macrophages (PVMs) and interferon responding genes, suggesting these cells to be macrophages (Fig. 3*A*). We further interrogated these clusters using PanglaoDB and established the top five cell types ranked by the odds ratio, suggesting that clusters 14 and 16 are macrophages (cluster 16 now deemed “macrophages”) and cluster 19 included T-cells and NK cells (Fig. 3*B*; Chen et al., 2013; Franzén et al., 2019). All three clusters showed decreasing cell population percentages from 24 h to 3 d in the IgG antibody condition, while clusters 14 and 19 increased from 24 h to 3 d in the anti-Aβ antibody condition (Fig. 3*C*).

10.1523/JNEUROSCI.1456-24.2024.f3-1 Figure 3-1 A. Percent change in number of immune cells per cluster from 24hr to 3day.B. Chord plot of TGFβ signaling at 3day in the anti-Aβ antibody and IgG antibody conditions.C. Sum of sending and receiving communication probability per cluster for TGFβ signaling at 3day between microglia and immune cells.. Download Multimedia/Extended Data, TIF file.

We further examined how communication differed between the microglia subtypes and other immune cells and asked whether this changed based on the time or type of antibody injected. Overall, microglia had generally more outgoing communication signaling than the immune cell clusters, with the most communication seen in the anti-Aβ antibody injection group at 24 h (Fig. 3*D,E*). Interestingly, cluster 14, the PVMs, had some of the highest incoming signaling strength at 24 h across both antibody groups; however, the response was stronger after anti-Aβ antibody injection. We next examined which signaling pathways the microglia clusters were using to communicate with the other immune cell clusters (Fig. 3*F,G*). Similar to the microglia-to-microglia communication, there were many more pathways that were significantly enriched after anti-Aβ antibody injection at 24 h compared with the IgG1 antibody injection at the same timepoint. The communication includes TGFβ, MHC-I, CD48, CCL, and SPP1 (Fig. 3*F*). Our results suggest that “TGFβ” signaling occurs uniquely in the anti-Aβ antibody condition at 24 h, whereas at 3 d, microglia-immune communications for “TGFβ” show significant enrichment in the IgG antibody injected condition (Fig. 3*F,G*). While almost all cell types were both sending and receiving TGFβ ligand–receptor communication at 24 h in both the anti-Aβ antibody and the IgG1 injection group, cluster 19, i.e., T/NK cells, was active in only sending TGFβ ligand–receptor communication in the anti-Aβ antibody condition (Fig. 3*H,I*). At 3 d after injection, microglia showed similar TGFβ communications between antibody conditions; however, cluster 14, perivascular macrophages, and cluster 19, T-cells and NK cells, became more active in the IgG1 antibody condition.

### Perivascular macrophage and nonparenchymal immune cell signaling is increased following anti-Aβ antibody injection

**Figure 4.**
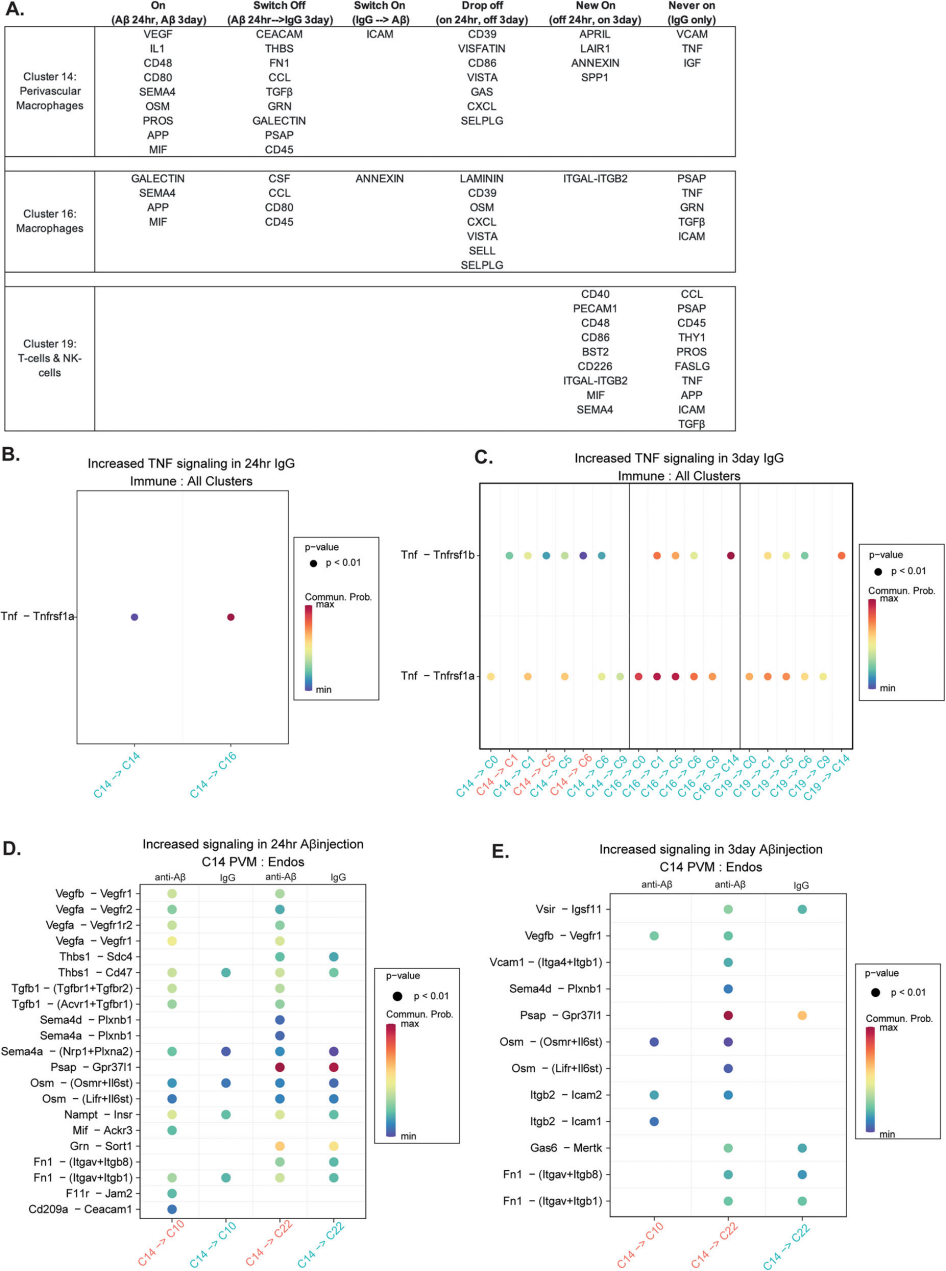
Nonmicroglial immune cells produce dynamic communication patterns at 24 h and 3 d. ***A***, A chart categorizing significant signaling pathways and their status at 24 h and 3 d for cluster 14 perivascular macrophages, cluster 16 macrophages, and cluster 19 T-cells and NK cells (Extended Data Fig. 4-1). ***B***, Significant ligand–receptor TNF signaling for immune clusters to all other clusters at 24 h. ***C***, Significant ligand–receptor TNF signaling for immune clusters to all other clusters at 3 d. ***D***, Significantly increased ligand–receptor signals for cluster 14 PVMs to endothelial cells clusters (10, 22) in the anti-Aβ antibody condition at 24 h. ***E***, Significantly increased ligand–receptor signals for cluster 14 PVMs to endothelial cells (clusters 10, 22) in the anti-Aβ antibody condition at 3 d.

Finally, we explored whether anti-Aβ antibody influences the outgoing communication patterns of the nonbrain-native immune cells. To identify significant signaling pathways, we examined the immune cell clusters' (14, 16, and 19) information flow to all other cell types separately. As we found in the microglial communication patterns, both cluster 14, PVMs, and cluster 16, macrophages, had many more active pathways at 24 h than at 3 d, suggesting an immediate, acute communication response in these cells after anti-Aβ antibody injection. Conversely, cluster 19, T-cells and NK cells, only showed communication at 3 d. To identify holistic changes that may be perturbed from anti-Aβ antibody exposure, we classified the enriched signaling pathways into the following: “On” (significant enrichment of the pathway in both anti-Aβ 24 h and anti-Aβ 3 d), “Switch Off” (switching from significant enrichment in anti-Aβ at 24 h to significant in IgG at 3 d), “Switch On” (switching from significant enrichment in IgG and not enriched in anti-Aβ at 24 h to significant in anti-Aβ and not in IgG at 3 d), “Drop-Off” (significant enrichment of the pathway in anti-Aβ at 24 h which becomes significant in neither condition at 3 d), “New On” (no significant enrichment at 24 h for either condition and significant enrichment for anti-Aβ at 3 d), and “Never On” (significant enrichment in the IgG condition but never the anti-Aβ condition; Fig. 4*A*). In the “Never On” category, TNF signaling was only enriched in the IgG antibody condition and never in the anti-Aβ antibody condition. In the IgG1 group, enriched TNF signaling from the immune cell clusters increased from 24 h to 3 d, showing that cluster 14, PVMs, communicate with themselves and with cluster 16, macrophages, at 24 h, while at 3 d, more instances of significant TNF communication emerge with multiple other microglia clusters at 3 d in the IgG antibody condition (Fig. 4*B,C*).

10.1523/JNEUROSCI.1456-24.2024.f4-1 Figure 4-1 A. Signaling pathways enriched in PVMs(C14, sending):All cell types(receiving) communication at 24hr in anti-Aβ (pink), and IgG (teal).B. Signaling pathways enriched in PVMs(C14, sending):All cell types(receiving) communication at 3day in anti-Aβ (pink), and IgG (teal).C. Signaling pathways enriched in macrophages(C16, sending):All cell types(receiving) communication at 24hr in anti-Aβ (pink), and IgG (teal).D. Signaling pathways enriched in macrophages(C16, sending):All cell types(receiving) communication at 3day in anti-Aβ (pink), and IgG (teal).E. Signaling pathways enriched in Tcells/NKcells (C19, sending):All cell types(receiving) communication at 3day in anti-Aβ (pink), and IgG (teal).F. Significantly increased MIF ligand-receptor signals for Immune cell clusters to All cell clusters in both injection conditions at 24hr.G. Significantly increased MIF ligand-receptor signals for Immune cell clusters (C14, C16, C19) to All cell clusters in both injection conditions at 3day.. Download Multimedia/Extended Data, TIF file.

Interestingly, multiple pathways were enriched at 24 h and 3 d with the anti-Aβ antibody condition (“On”), including SEMA4, APP, and MIF, across both cluster 14, PVMs, and cluster 16, macrophages. MIF signaling has been implicated in Aβ-mediated inflammation, and so we examined which of our clusters and ligand–receptor pairs are responsible for the MIF signaling enrichment. MIF signaling from each of the three immune clusters utilized CD74 and CD44 to signal to microglia DAM, cluster 6, at 24 h. The MIF-Ackr3 ligand–receptor pair was significantly enriched for the anti-Aβ antibody injection condition in communication with cluster 11, OPCs, at both 24 h and 3 d. In PVMs specifically, there were many signaling pathways that were significantly enriched for the anti-Aβ antibody injection condition at both the 24 h and 3 d timepoints (Fig. 4*A*). Due to the PVM's proximity to the vasculature and previously established importance in ARIA, we probed further to find out which pathways PVMs were utilizing to communicate with endothelial cells. At 24 h, there were multiple VEGF ligand–receptor pairs that were exclusively significant in the anti-Aβ antibody condition at 24 h, including Vegfb-Vegfr1, which was also significant for the anti-Aβ antibody condition at 3 d (Fig. 4*D,E*). SEMA4 signaling was also enriched in the anti-Aβ antibody condition, with receptor Plxnb1 on endothelial cells having a similar vascular remodeling effect as the VEGF family.

## Discussion

With anti-Aβ targeting antibodies in use for immunotherapy to treat AD, it is imperative to understand the interaction of the antibodies with the cerebral milieu. Due to a remaining lack of understanding surrounding the basic cellular responses to anti-Aβ antibodies, further refinement of these biological approaches to treat AD to increase safety and efficacy remains difficult. The first step in establishing the cellular responses is to understand the acute cellular responses upon exposure of the brain to anti-Aβ antibody and how this differs from exposure to an irrelevant antibody. Previous studies have identified both microglial-mediated and nonmicroglial-mediated clearance of Aβ after antibody exposure, suggesting that there may be a temporal component by which the brain relies on microglia to clear Aβ. Here, we evaluate the communication patterns between microglia and noncerebral-native immune cells 24 h or 3 d following intracranial injection of anti-Aβ antibody and control IgG antibody. Our work shows anti-Aβ antibody-specific differences that are not present in the nonspecific IgG antibody condition, highlighting increased acute communication through CD48, PD-L1, CCL, and SPP1 within microglia, microglia communicating to nonparenchymal immune cells via TGFβ, a blunted response of anti-Aβ antibody exposed cells to TNF signaling, and increased perivascular macrophage communication to endothelial cells.

Our single-cell sequencing revealed 23 distinct cell clusters, six of which were categorized as microglia (clusters 0, 1, 5, 6, 7, 9, and 12). With the substates of these microglial clusters identified in Figure 2, we were surprised to see modest changes in cluster 6 DAM cells between the anti-Aβ antibody and IgG antibody conditions between 24 h and 3 d (anti-Aβ, from 5.5% to 6.3%; IgG, from 6.7% to 4.6%). As these are classically thought to be the microglia that respond to a plaque-rich environment, these changes suggest that it might take days for DAM cells to mount a robust response. Interestingly, there was a decrease in cluster 6 DAM cells in the IgG condition over time, while there was a slight increase in the anti-Aβ antibody condition (Fig. 2*B*). Moreover, the cluster 5 “pre-DAM” cells had a greater change in the anti-Aβ antibody exposed condition, growing from 6.6% to 10.0% of the total cell population collected (compared with the IgG condition decrease which was from 6.4% to 5.8%). Due to the acute timepoints in this study, it can be predicted that these microglia are in the process of “activating” and transcriptionally increasing proinflammatory transcripts for protein production.

Among the communication differences measured by the number of interactions, and the interaction weight, it was clear that microglia undergo significant transcriptional changes due to anti-Aβ antibody as early as 24 h postinjection (Fig. 1*E,F*). We were surprised to see the number of pathways that were significantly enriched for microglia-to-microglia communication in the anti-Aβ antibody compared with the IgG antibody condition, including CD48-CD244a. CD48 is part of the signaling lymphocyte activation marker (SLAM) family and has been previously shown to have a role in immunomodulation of cells in the hematopoietic linage (B-cells, T-cells, macrophages); however, gene expression databases of the brain also show expression in microglia (brainrnaseq.org, betsholtzlab.org; Elishmereni and Levi-Schaffer, 2011; McArdel et al., 2016). CD48 expression is known in the autoimmune field to recruit nonparenchymal immune cells through cell adhesion mechanisms and proinflammatory signaling; however, the specific effects of both ligand and receptor binding on microglia are unknown. Interestingly, one study evaluated CD48 together with PD-L1, an immune checkpoint mechanism in glioma, suggesting that these two upregulated signaling pathways with anti-Aβ antibody exposure may trigger acute immunomodulatory response first (Zou et al., 2019). Furthermore, one study revealed that when PD1, a PD-L1 receptor, is reduced, there is an increase in amyloid pathology (Kummer et al., 2021). CCL signaling in microglia showed a dichotomous enrichment in microglia clusters between the sending and receiving signals, with sending CCL signaling primarily originating from pre-DAM and DAM clusters and receiving receptors in the homeostatic clusters. This specific breakdown of CCL communication highlights the possible engagement of “activated” microglia in recruiting nonresponsive microglia to another cell state. Similar to this, SPP1 signaling is primarily propagated by cluster 6 DAM cells and proliferative microglia, aligning with a previously demonstrated increased expression of SPP1 in plaque-associated microglia (Van Hove et al., 2019; De Schepper et al., 2023); however, the role of this signaling in Aβ clearance is not fully understood. Additionally, our analysis shows differences in these signaling pathways between clusters, with clusters 5 and 6 being the most active in this communication; however, there were also differences between the signaling, ligand bearing and receiving, receptor bearing, and cell populations, implicating a dual response is needed for proper completion of signaling.

Given our growing understanding that the brain is not devoid of nonparenchymal immune cells, we also evaluated the communication between microglia and other non-native immune cells. Firstly, we would like to address the classification of “nonparenchymal immune cells,” of which we placed PVMs, macrophages, and circulating T-cell clusters. While microglia are typically considered the only “true” CNS parenchymal macrophage, there are studies uncovering yolk sac-derived boarder-associated macrophages, blurring the line of the “brain-native” macrophage status (Jordan and Thomas, 1988). Our goal here was to separate the microglia and other immune cells with as much discretion as possible on the basis of function and location, leaving the nomenclature as “microglia” and its several subtypes and “nonparenchymal immune cells” and its subtypes. Much of the communication that was significant in microglia–microglia cross talk was also seen in the three nonparenchymal “immune” cell clusters (14 PVMs, 16 macrophages, 19 T-cells/NK cells, Fig. 2*E*). We saw that of the non-native immune cells, there is generally more communication identified in the PVMs, for both outgoing and incoming signaling (Fig. 3*D,E*). The signaling from and to microglia was greater on average. Interestingly, TGFβ signaling was the topmost enriched pathway for micro-to-immune communication in the anti-Aβ antibody injected condition at 24 h; however, at 3 d this signaling was enriched in the IgG1 antibody condition (Fig. 3*F,G,H,I*).

Previous studies have shown that TGFβ signaling is involved in encouraging microglia Aβ plaque clearance; however, this was evaluated with TGFβ1 overexpression in astrocytes specifically (Wyss-Coray et al., 2001). Interestingly, while the study showed a decrease in parenchymal plaque load, there was an increase in CAA. It has also been shown that in aged individuals, TGFβ is often elevated. Others have proposed that in a chronically inflamed state, microglia may become less responsive to TGFβ signaling (Von Bernhardi et al., 2015). It is possible that the triggering of TGFβ signaling may influence microglia to “clear” plaques from the parenchyma to the vasculature, instead of phagocytosing (Li and Barres, 2018). Furthermore, it was interesting that TGFβ signaling was not enriched in the anti-Aβ antibody condition in either cluster 16 macrophage or cluster 19 T-cell/NK cell communication to other cell types (Fig. 4*A*). Nonetheless, it is likely that TGFβ communication from other cell types (i.e., previously mentioned microglia:immune, neurons and astrocytes) play a role; however, the findings here show specific ligand–receptor pairings between brain-native microglia and macrophage cells, suggesting a more complicated mechanism at play.

Due to the previous indication of the involvement of PVMs at vessels associated with amyloid and increased leakage, we focused specifically on the communication patterns of PVMs with endothelial cells (Taylor et al., 2023). Although the previous findings were from chronic, systemic administration studies of anti-Aβ immunotherapy, we were curious whether there was an acute response of PVMs that may affect the vasculature. Results showed enrichment of multiple ligand–receptor pairs in the anti-Aβ antibody condition at both 24 h and 3 d. VEGF signaling appears enriched only in the anti-Aβ conditions (Fig. 4*D,E*). Taylor et al. also showed an increase in Prussian blue leakage associated with non-native immune cells, such as macrophages, leukocytes, and monocytes. These data collectively suggest that non-native immune cells may play a critical role in cerebrovascular leakage, with signaling starting as early as 24 h after anti-Aβ antibody exposure.

While informative, our study was a directed attempt to understand the immediate transcriptional response of immune cells to anti-Aβ antibody exposure. In this design, we use intracranial injections of 6E10, an IgG1 antibody against residues 1–16 of Aβ. This antibody has also been known to bind the APP sequence, and although not identical to the antibody epitopes and isotypes in use in the clinic and in clinical trials, it is still in the N-terminal region of Aβ akin to aducanumab and lecanemab. While the process of intracranial injection is confounded by the injection procedure into the brain parenchyma, which we controlled by utilizing an IgG1 control, our goal was to understand the acute central immune responses when antibody is inside the brain.

As ARIA and new disease-modifying anti-Aβ antibody therapies are bringing this type of work to the forefront, it is important to consider the multiple factors that may influence the immune response. The scope of this study, while revealing, also neglects the contribution of CAA versus parenchymal Aβ in the immune responses presented. Research from the early 2000s as well as current findings have suggested that CAA contributes to both immune response and downstream cerebrovascular dysfunction. For example, in 2013, Carare and colleagues found that ovalbumin, IgG, and complement C3 colocalized in the basement membrane of artery walls 24 h after challenge with the ovalbumin antigen; this was associated with significantly reduced drainage of dextran in immunized mice (Carare et al., 2013). Their interpretation was that when immune complexes form in association with basement membranes of cerebral arteries, they interfere with perivascular drainage of solutes from the brain. Furthermore, they suggested that the immune complexes formed during anti-Aβ immunotherapy in individuals with AD may similarly impair perivascular drainage of soluble Aβ and increase the severity of CAA. Previous publications using both active and passive anti-Aβ immunotherapy in mouse models and in human clinical trials provide support for this hypothesis (Wilcock et al., 2004, 2007; Masliah et al., 2005; Boche et al., 2008). In 2004, Masliah et al. published a case report from the active Aβ vaccination AN1792 clinical trial, observing that the frontal cortex was void of Aβ plaques; however, there were abundant Aβ-immunoreactive macrophages and prominent CAA present in the frontal cortex with alone with significant tangles. In 2004, Wilcock et al. reported that passive anti-Aβ immunotherapy depleted amyloid plaques with a corresponding CAA and microhemorrhage over a 3-month period (Wilcock et al., 2004). In 2007, Wilcock et al. performed an active anti-Aβ immunotherapy study where they once again saw an increase in CAA and microhemorrhages in response to anti-Aβ antibodies (Wilcock et al., 2007). Finally, in 2008, James Nicoll and colleagues reported that compared with nonimmunized controls, the group of immunized patients had approximately 14 times as many blood vessels containing Aβ_42_ in the cerebral cortex and seven times more in the leptomeninges; among the affected blood vessels in the immunized cases, most of them had full thickness and full circumference involvement of the vessel wall in the cortex, and in the leptomeninges (Boche et al., 2008). While these publications were from over 15 years ago, there are still many remaining questions surrounding the influence of chronic anti-Aβ antibodies on the immune system and their impact on the cerebrovasculature.

Our work showed immediate changes in microglial and other immune cells’ molecular profiles in response to anti-Aβ antibody exposure when compared with a control IgG antibody. These findings suggest early activation states prompted by the Aβ-targeting aspect of the antibody that triggers increased microglial communications through proinflammatory signaling and strong communication with other non-native immune cells. Although acute, these findings suggest there is a complicated temporal network of communication between microglia and nonmicroglial immune cells that is induced by anti-Aβ antibody exposure, and further research is needed to parse out the beneficial and detrimental roles of this neuroinflammation and possible downstream vascular deficits.
