# Supplementary figures and images for: Acute Communication Between Microglia and Nonparenchymal Immune Cells in the Anti-Aβ Antibody-Injected Cortex
Source: J Neurosci. 2024 Dec 31;45(5):e1456242024. doi: 10.1523/JNEUROSCI.1456-24.2024 (PMC11780351; doi:10.1523/JNEUROSCI.1456-24.2024)

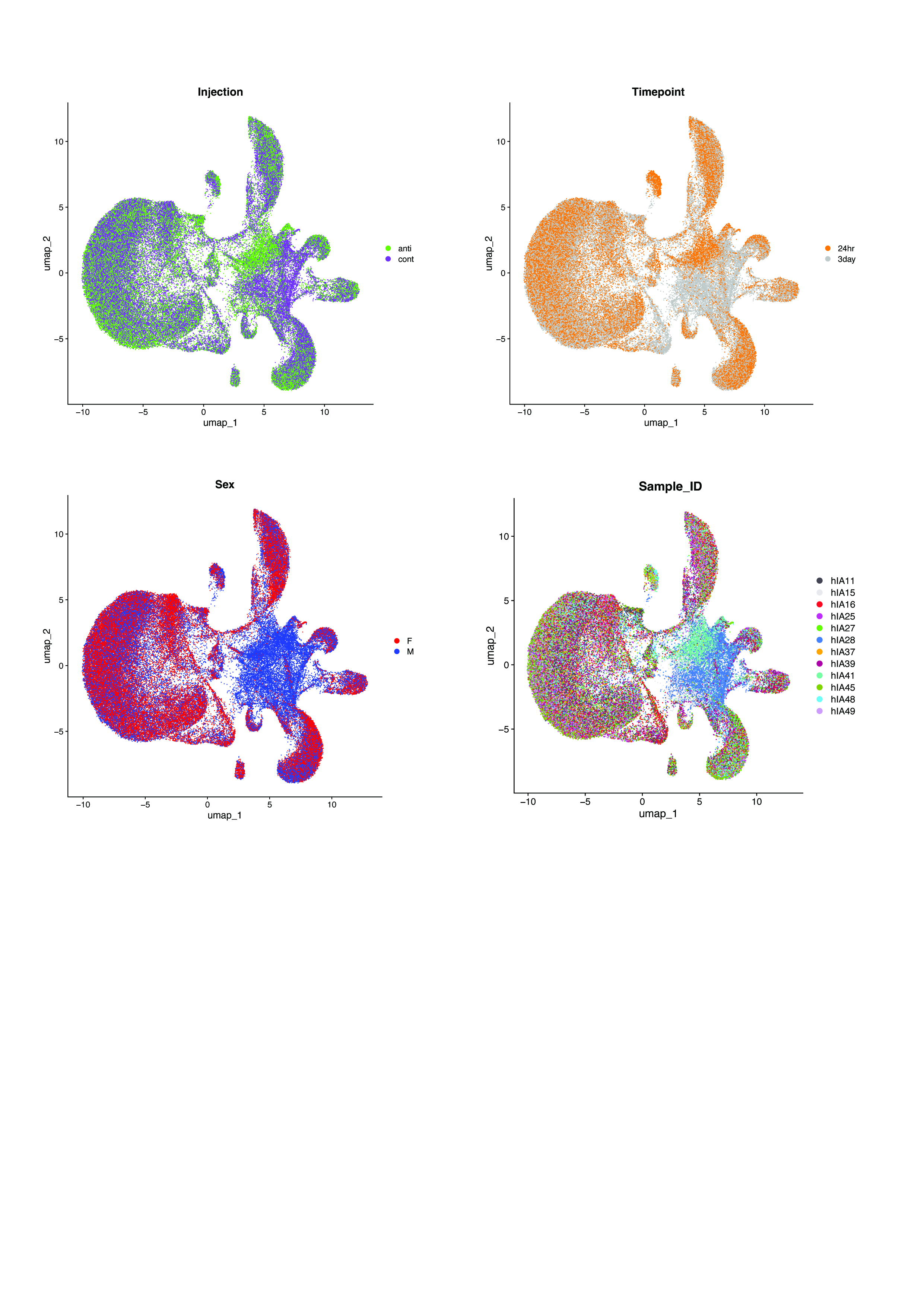

Supplement: Figure 1-1 — UMAP graphs colored for injection type, timepoint, sex, and sample ID. Download Multimedia/Extended Data, TIF file. [file jneuro-45-e1456242024-s002.tif]

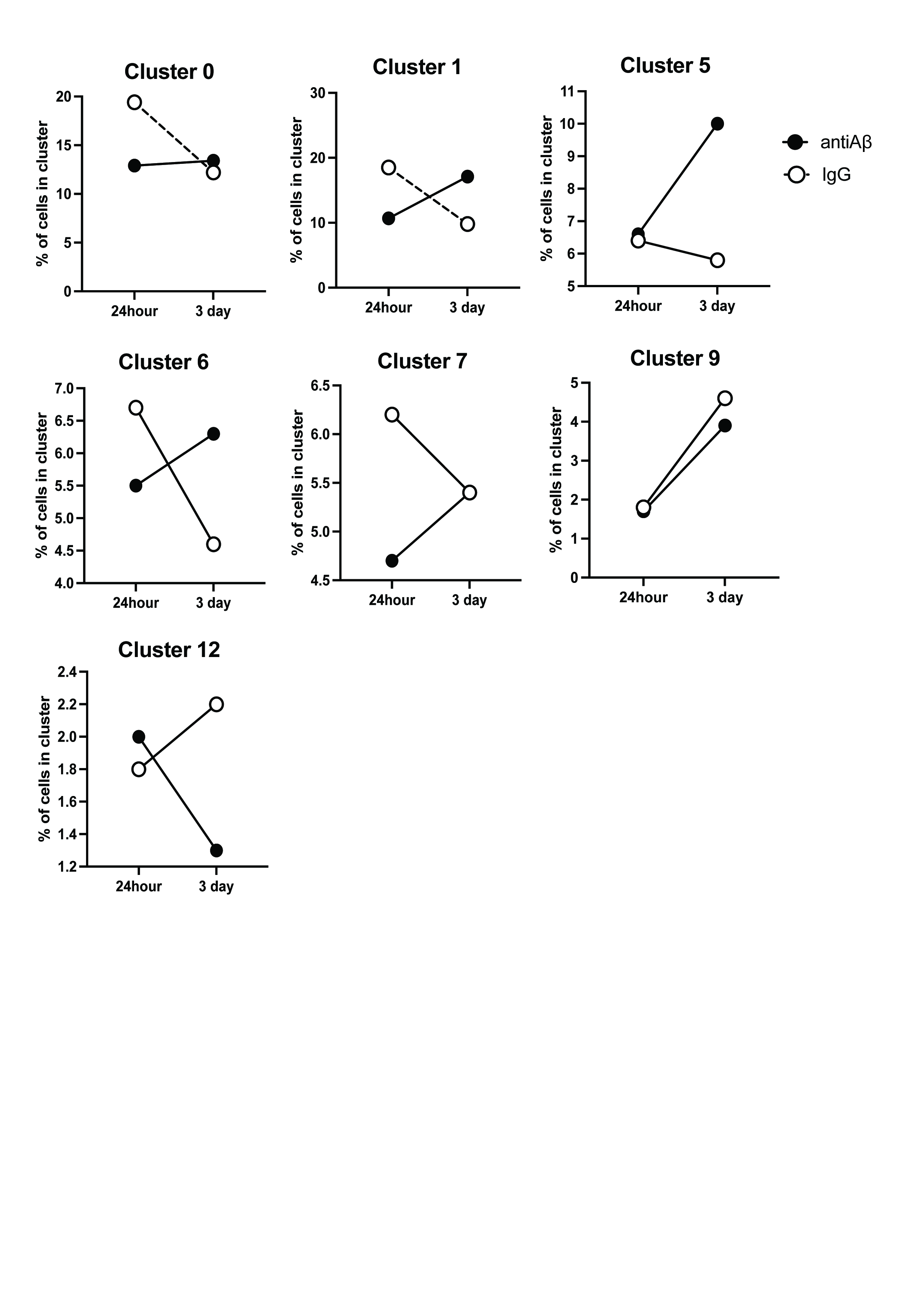

Supplement: Figure 2-1 — Percentage of cells in microglia clusters at 24hrs and 3days. Download Multimedia/Extended Data, TIF file. [file jneuro-45-e1456242024-s004.tif]

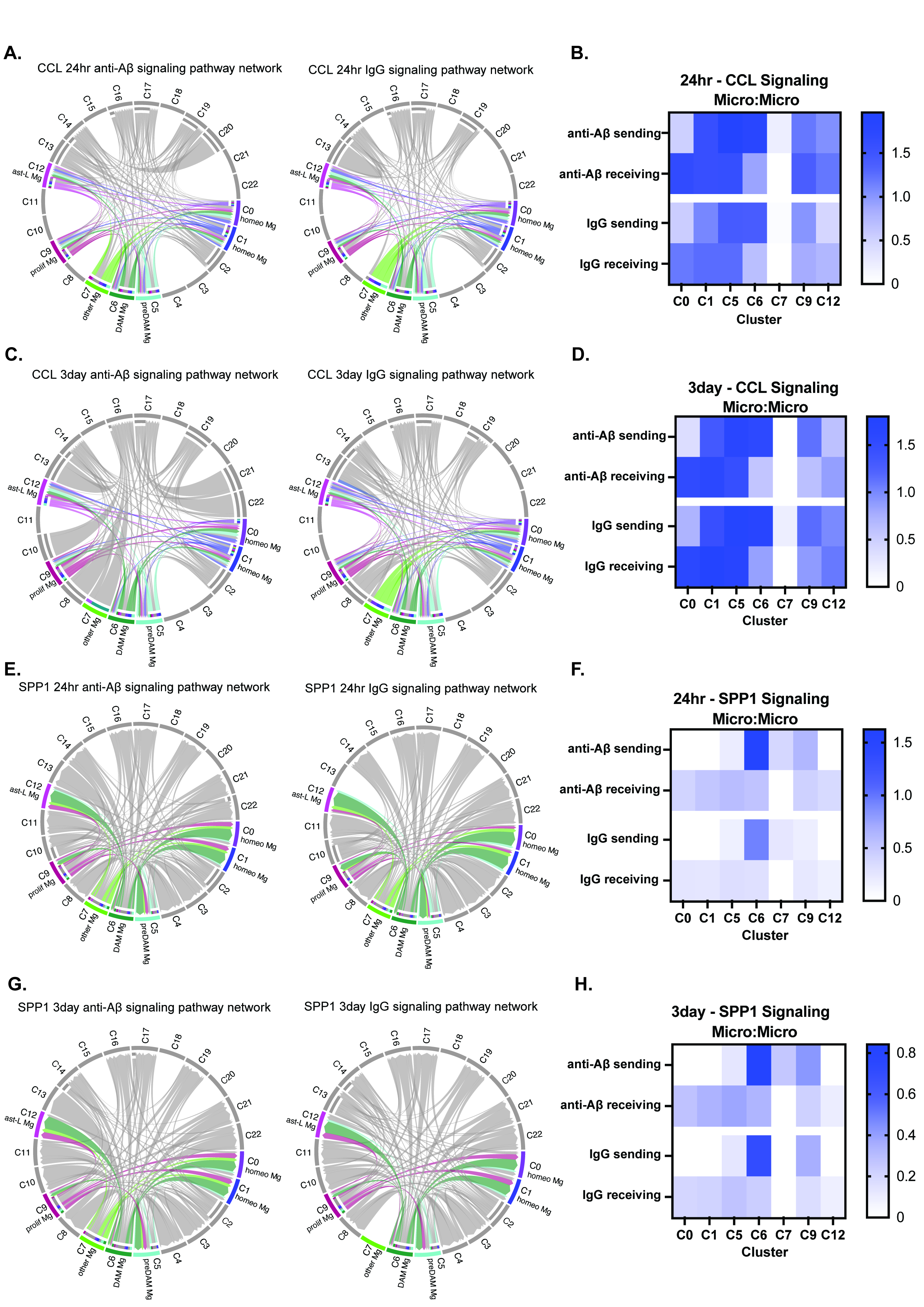

Supplement: Figure 2-2 — A. Chord plot of CCL signaling at 24hr in the anti-Aβ antibody and IgG antibody conditions.B. Sum of sending and receiving communication probability per cluster for CCL signaling at 24hr between microglia.C. Chord plot of CCL signaling at 3day in the anti-Aβ antibody and IgG antibody conditions.D. Sum of sending and receiving communication probability per cluster for CCL signaling at 3day between microglia.E. Chord plot of SPP1 signaling at 24hr in the anti-Aβ antibody and IgG antibody conditions.F. Sum of sending and receiving communication probability per cluster for SPP1 signaling at 24hr between microglia.G. Chord plot of SPP1 signaling at 3day in the anti-Aβ antibody and IgG antibody conditions.H. Sum of sending and receiving communication probability per cluster for SPP1 signaling at 3day between microglia. Microglia communications are colored according to cluster sending (ligand), and non-microglia communications are in grey. Download Multimedia/Extended Data, TIF file. [file jneuro-45-e1456242024-s005.tif]

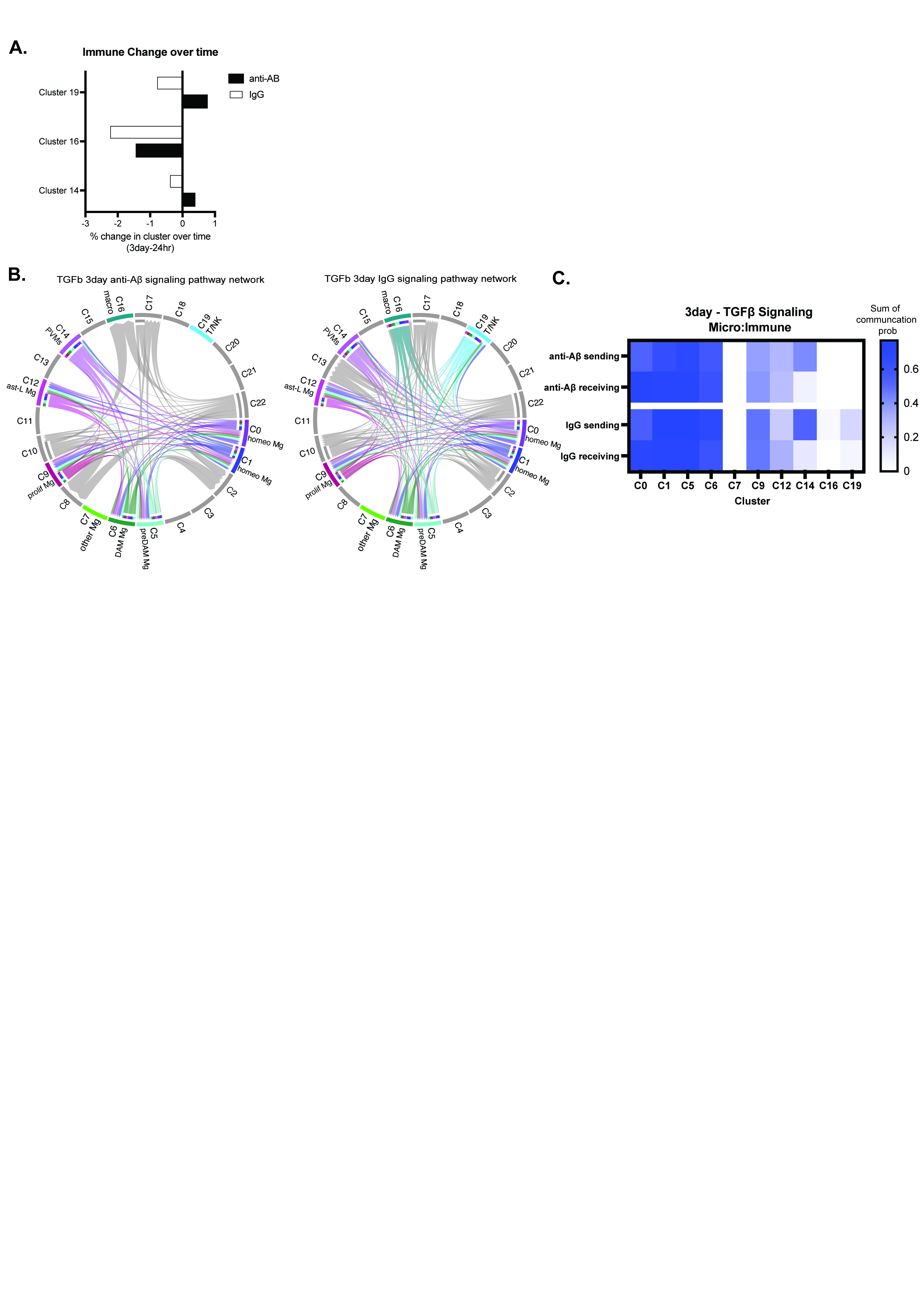

Supplement: Figure 3-1 — A. Percent change in number of immune cells per cluster from 24hr to 3day.B. Chord plot of TGFβ signaling at 3day in the anti-Aβ antibody and IgG antibody conditions.C. Sum of sending and receiving communication probability per cluster for TGFβ signaling at 3day between microglia and immune cells.. Download Multimedia/Extended Data, TIF file. [file jneuro-45-e1456242024-s006.tif]

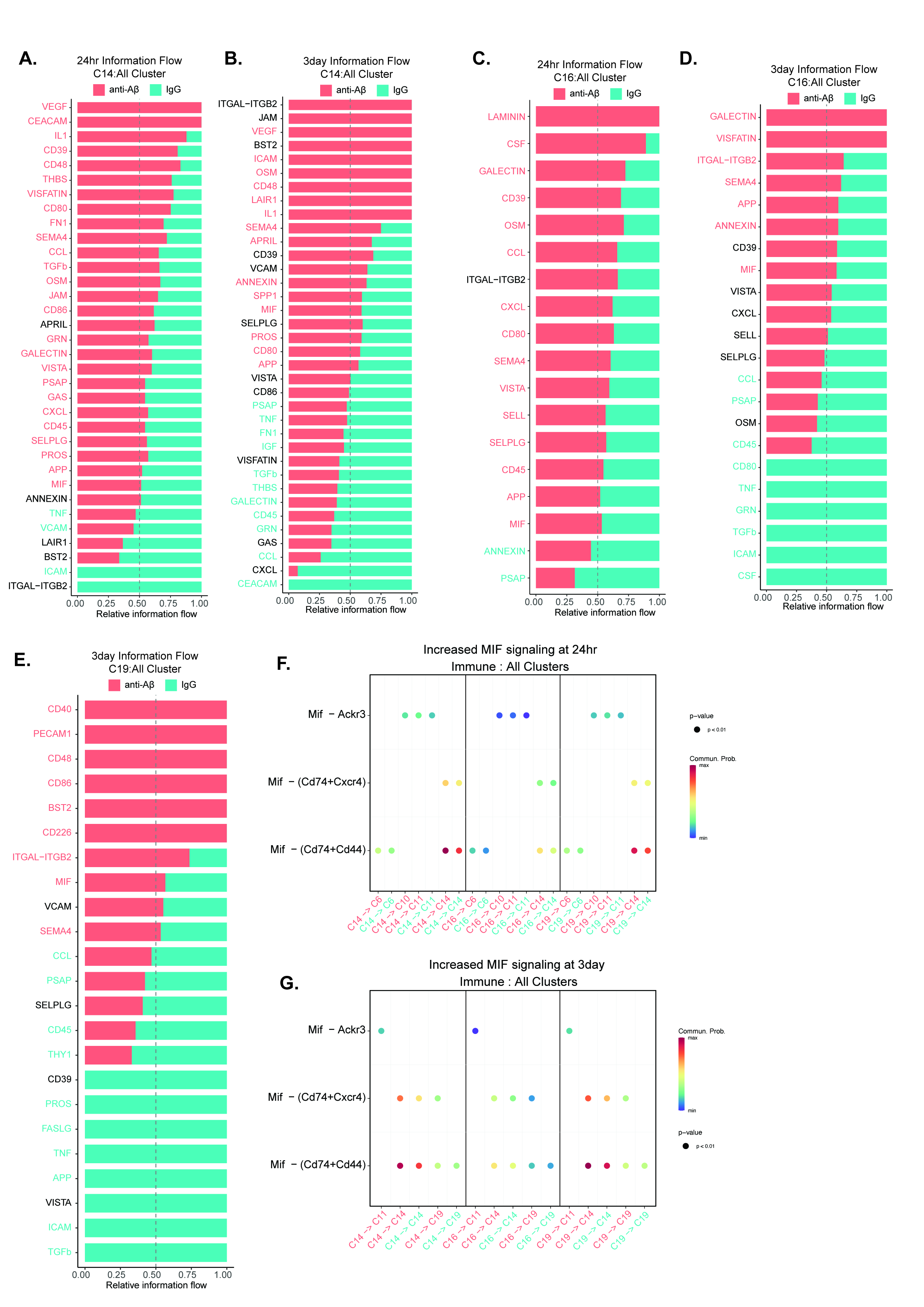

Supplement: Figure 4-1 — A. Signaling pathways enriched in PVMs(C14, sending):All cell types(receiving) communication at 24hr in anti-Aβ (pink), and IgG (teal).B. Signaling pathways enriched in PVMs(C14, sending):All cell types(receiving) communication at 3day in anti-Aβ (pink), and IgG (teal).C. Signaling pathways enriched in macrophages(C16, sending):All cell types(receiving) communication at 24hr in anti-Aβ (pink), and IgG (teal).D. Signaling pathways enriched in macrophages(C16, sending):All cell types(receiving) communication at 3day in anti-Aβ (pink), and IgG (teal).E. Signaling pathways enriched in Tcells/NKcells (C19, sending):All cell types(receiving) communication at 3day in anti-Aβ (pink), and IgG (teal).F. Significantly increased MIF ligand-receptor signals for Immune cell clusters to All cell clusters in both injection conditions at 24hr.G. Significantly increased MIF ligand-receptor signals for Immune cell clusters (C14, C16, C19) to All cell clusters in both injection conditions at 3day.. Download Multimedia/Extended Data, TIF file. [file jneuro-45-e1456242024-s007.tif]
